# Editorial: Epigenetic Modifications in Mesothelioma

**DOI:** 10.3389/fonc.2021.650136

**Published:** 2021-02-03

**Authors:** Yuen Yee Cheng, Man Lee Yuen, Hongchuan Jin

**Affiliations:** ^1^ Asbestos Diseases Research Institute, The University of Sydney, Sydney, NSW, Australia; ^2^ Biomedical Research Centre, Sir Runrun Shaw Hospital, Zhejiang University, Hangzhou, China

**Keywords:** mesothelioma, microRNA, DNA methylation, epigenetic, diagnosis

## Introduction

We are pleased to announce the publication of this Research Topic focusing on epigenetic modifications in mesothelioma. Epigenetic modification has been investigated for the past two decades. It is a key process of cellular development as well as carcinogenesis. Mesothelioma is an aggressive cancer caused by asbestos exposure and currently has very limited effective treatment options. Epigenetic modification in mesothelioma is an emerging field which will provide fundamental information for diagnosis and treatment of mesothelioma. We believe that, as for other cancers, if we can detect this cancer at an as early stage as possible, then we can significantly increase survival time as well as select suitable treatments for the patients. Currently, misdiagnosis and delayed treatment jeopardises patient survival and quality of life.

Mesothelioma is one of the most difficult cancers to diagnose, due to symptoms closely resembling lung cancer and other lung diseases. The most discussed topics in this Research Topic are biomarkers and how to diagnose mesothelioma. Rozitis et al. discuss the current methods used to diagnose mesothelioma. The take home message of this review is that at least nine immunohistochemistry biomarkers are used in the clinical setting to facilitate mesothelioma diagnosis. A tissue or cytology sample is normally required for definitive diagnosis. The authors mention the necessity to further develop epigenetic biomarkers to facilitate clinical diagnosis. A novel class of biomarker - the “circular RNA” is also discussed in the review. The review provides the basis of how mesothelioma is currently diagnosed and reviews emerging epigenetic biomarkers in mesothelioma. Although DNA methylation is the most discussed epigenetic biomarker in other cancers, microRNA represents the most interesting for mesothelioma researchers. Tomasetti et al. focus on microRNA as an early diagnosis biomarker for mesothelioma. The authors cover how reactive oxygen species (ROS) induced from asbestos can cause epigenetic alterations and aberrant gene expression. MicroRNAs are stable and amenable for treatment development. Unlike protein-coding genes in gene replacement therapy, microRNAs are small and more easily to restore their “cancer killer” functions. More importantly, the authors discuss microRNA replacement therapy in mesothelioma for which there has been one on-going clinical trial. We believe this review will trigger further development of microRNA replacement to treat mesothelioma. Biomarkers that facilitate other treatment options leading to better outcomes in mesothelioma are another emerging area of discussion. Yoshikawa et al. research the benefits of epigenetic alterations and its potential in advancing the response to immune checkpoint PD-1 inhibitor nivolumab. The authors describe recent knowledge on epigenetic alterations, which identify potential candidate therapeutic targets and immunotherapeutic regimens under development for MPM. Being a disease that is difficult to diagnose, researchers have tried various biomarkers that are anticipated to improve mesothelioma diagnosis. CDKN2A fluorescence in-situ hybridization is one of the biomarkers used in a clinical setting to facilitate differential mesothelioma diagnosis. Cheng et al. re-validate CDKN2A and MTAP, which are usually co-deleted in mesothelioma, in a large cohort of MPM patient samples. The authors promote the use of droplet digital PCR (ddPCR) as an alternative to study these two biomarkers in mesothelioma. Amongst all the epigenetic biomarkers discussed in the special edition, circulating biomarker is frequently explored because of its advantage in requiring less-invasive detection routes. With the development of new detection methods, biomarkers that provide high sensitivity and specificity can easily be further investigated for their clinical applications. Epigenetic biomarkers such as DNA methylation, microRNA and non-coding RNA have high potential to provide sensitive and specific detection of mesothelioma as well as providing earlier possible detection.

Epigenetic regulation is a rewarding area of study, as it is involved in almost all developmental processes including the early stage of cancers. Different epigenetic signatures can be used to identify different stages of cancer development. This makes it relatively useful for further development for early detection of mesothelioma. Due to a long latency period from asbestos exposure to development of mesothelioma, most patients are diagnosed at a late stage of the disease when very limited treatment options are available for effective outcomes. Early detection utilizing epigenetic biomarkers to identify mesothelioma will be beneficial for this deadly disease. Epigenetic biomarkers have great potential to become more conclusive diagnostic and prognostic biomarkers for mesothelioma. The development of new technologies will also largely facilitate biomarker detection sensitivity. However, further research is required in this field to ensure the widespread application of epigenetic biomarkers in clinical settings.

In conclusion, epigenetic regulation plays a major role in mesothelioma and holds great potential to become clinically relevant in mesothelioma. More studies are required to further develop and validate the clinical applications of epigenetic biomarkers in mesothelioma.

## Author Contributions

YC and HJ contributed equally to this editorial. MY contributed to the final edit of this editorial. All authors contributed to the article and approved the submitted version.

## Conflict of Interest

The authors declare that the research was conducted in the absence of any commercial or financial relationships that could be construed as a potential conflict of interest.

